# Footwear designed to enhance energy return improves running economy compared to a minimalist footwear: does it matter for running performance?

**DOI:** 10.1590/1414-431X202010693

**Published:** 2021-03-15

**Authors:** R.C. Dinato, R. Cruz, R.A. Azevedo, J.S. Hasegawa, R.G. Silva, A.P. Ribeiro, A.E. Lima-Silva, R. Bertuzzi

**Affiliations:** 1 Grupo de Estudo em Desempenho Aeróbio, Escola de Educação Física e Esporte, Universidade de São Paulo, São Paulo, SP, Brasil; 2 Centro de Desportos, Departamento de Educação Física, Universidade Federal de Santa Catarina, Florianópolis, SC, Brasil; 3 Human Performance Laboratory, Faculty of Kinesiology, University of Calgary, Calgary, Canada; 4 Departamento de Pós-Graduação em Ciências da Saúde, Laborátorio de Biomecânica e Reabilitação Musculoesquelética, Universidade de Santo Amaro, São Paulo, SP, Brasil; 5 Grupo de Pesquisa em Performance Humana, Departamento Acadêmico de Educação Física, Universidade Tecnológica Federal do Paraná, Curitiba, PR, Brasil

**Keywords:** Running shoes, Oxygen uptake, Oxygen cost, Running time-trial, Endurance performance

## Abstract

The present study compared the effects of a footwear designed to enhance energy return (thermoplastic polyurethane, TPU) *vs* minimalist shoes on running economy (RE) and endurance performance. In this counterbalanced and crossover design study, 11 recreational male runners performed two submaximal constant-speed running tests and two 3-km time-trials with the two shoe models. Oxygen uptake was measured during submaximal constant-speed running tests in order to determine the RE at 12 km/h and oxygen cost of running (CTO_2_) at individual average speed sustained during the 3-km running time-trials wearing either of the two shoes. Our results revealed that RE was improved (2.4%) with TPU shoes compared with minimalist shoes (P=0.01). However, there was no significant difference for CTO_2_ (P=0.61) and running performance (P=0.52) comparing the TPU (710±60 s) and the minimalist (718±63 s) shoe models. These novel findings demonstrate that shoes with enhanced mechanical energy return (i.e. TPU) produced a lower energy cost of running at low (i.e., 12 km/h) but not at high speeds (i.e., average speed sustained during the 3-km running time-trial, ∼15 km/h), ultimately resulting in similar running performance compared to the minimalist shoe.

## Introduction

Running endurance performance has been traditionally associated with several physiological variables, including running economy (RE) (1–5). Individuals with superior RE, defined as the steady-state oxygen uptake at submaximal running speeds (4), are able to sustain higher exercise intensities and/or maintain the same exercise intensity for a longer period of time compared to their counterparts with poorer RE (6). In order to acutely improve RE, previous studies have suggested that footwear characteristics could play a significant role on the energy cost of running. For example, minimalist shoes (i.e., with reduced shoe mass and heel drop) results in significant improvements in RE (1-4%) compared to conventional shoes (i.e., with ethylene vinyl acetate midsole) (7–9). This enhanced RE with minimalist shoes has been associated with a greater mechanical action of the longitudinal and transverse arches of the foot, which are capable of restoring/returning approximately 17% of the mechanical energy temporarily stored at each step taken (10), thus acutely improving the RE at submaximal running.

Other studies have demonstrated that midsole characteristics can also enhance RE (11,12). For instance, Wunsch et al. (13) showed that a leaf spring-structured midsole acutely improved RE (∼1%), probably by changes in spatio-temporal variables. More recently, a new midsole material composed by thermoplastic polyurethane (TPU) has been used to enhance energy return during running (14). The TPU appears to reduce oxygen cost of running by increasing the returned mechanical energy from the shoe midsole material. In fact, Sinclair et al. (15) have shown that running with a footwear with a midsole composed by TPU exhibits a better RE (4.1%) compared to a conventional running shoe. Given that RE contributes to the success in endurance running events, they concluded that footwear with a TPU midsole could lead to better running performance, probably due to the beneficial effects on RE.

Despite the remarkable findings from previous studies showing that both TPU and minimalist shoes can reduce the oxygen cost of running compared with conventional shoes (7,15), it is still unknown whether there is a superior ability between these two models to translate the greater RE into better running performance. This occurs mainly because the current evidence is limited to analyzing the changes in RE (15–17), without necessarily examining whether improved RE translated into better running performance. This is particularly relevant because previous findings have indicated that improvement in RE does not necessarily result in a better running performance (18). From the practical perspective, this information might be helpful to sports physiologists to better select sport shoes for competition and training. Therefore, the present study aimed to: i) compare the effects of TPU and minimalist shoes on the oxygen cost of running; and ii) analyze the effect of a possible reduced oxygen cost of running mediated by these shoes on running performance.

## Materials and Methods

### Participants

The sample size required was estimated using the G*power software (version 3.1.9.2, Germany), with data from a previous investigation that analyzed the effect of different midsole characteristics (TPU *vs* conventional) on RE (15). A sample size of five participants was estimated to achieve statistically significance in RE, for an expected effect size of 1.92 and power of 0.8 with an alpha level of 0.05. In order to improve statistical power, eleven recreational male runners volunteered to participate in this study. Participants were engaged in local competitions and their best performances in 10-km race times ranged from 35 to 45 min. All participants performed only low-intensity continuous aerobic training (50-70% maximal oxygen uptake, O_2_max) and were instructed to maintain a similar aerobic training during the experimental period. The exclusion criteria were: i) exhibited a forefoot contact running technique; ii) use of dietary supplement; iii) neuromuscular disorders; and iv) cardiovascular dysfunctions. The participants received a verbal explanation about the possible benefits, risks, and discomforts associated with the study and signed a written informed consent before participating in the study. The study was conducted according to the Declaration of Helsinki and approved by the Research Ethics Committee of the School of Physical Education and Sport of the University of São Paulo (protocol number 37502714.8.0000.5391).

### Experimental design

Participants visited the laboratory on four separate occasions at least 48 h apart and within a 4-week period. On the first visit, after the anthropometric measurements, the participants performed an incremental test to exhaustion in order to determine their O_2_max wearing their own running shoes. During second and third visits, the participants performed, in a counterbalanced design, two 3-km running time-trials (3-km TT) on an outdoor 400-m track wearing either the TPU or minimalist shoes. On the fourth visit, the oxygen uptake was measured, in a counterbalanced design, during constant-speed running tests performed at 12 km/h (i.e., RE) and at individual average speed sustained during the 3-km TT (i.e., CTO_2_), wearing either the TPU or minimalist shoes. Between each experimental condition (speed *vs* shoes), the participant rested for 10 min. Prior to the experimental laboratory and field tests, the participants were submitted to a 3-min familiarization run wearing either the TPU or minimalist shoes, as previously described (8). The tests were performed during the preparatory training period, at the same time of the day, and at least 2 h after the last meal. The participants were instructed to record their diet 24 h before the first experimental session and to repeat it prior to the subsequent experimental sessions. They were also asked to refrain from any exhaustive or unusual exercise during the experimental period.

### Anthropometric measurements

An experienced investigator performed the anthropometric measurements according to the procedures described by Norton and Olds (19). Participants were weighed to the nearest 0.1 kg using an electronic scale (Filizola, model ID 1500, Brazil). Height was measured to the nearest 0.1 cm using a stadiometer. Skinfold thickness was measured to the nearest 0.2 mm at six body sites (triceps brachial, suprailiac, abdominal, chest, subscapular, and anterior thigh) using a Harpenden caliper (West Sussex, UK). The mean of three values was used for further analysis. Body density and body fat were estimated by the equations from Jackson et al. (20) and Brozek et al. (21), respectively.

### Incremental maximal test

The incremental maximal test was performed on a motor-driven treadmill (model TK35, CEFISE, Brazil). All participants were requested to wear their favorite running shoes for this test. After a 3-min warm-up at 8 km/h, the speed was increased by 1 km/h every minute until participants were unable to maintain the required running speed. The subjects received strong verbal encouragement to continue as long as possible. Oxygen uptake (V·O_2_), carbon dioxide production, and ventilation were measured breath-by-breath using an automatic metabolic cart (Cortex, Metalyzer 3B, Germany) and subsequently averaged over 30-s intervals throughout the test. Before each test, the metabolic cart was calibrated using a 3-L syringe and a standard gas of known O_2_ (12%) and CO_2_ (15%) concentrations. The O_2_max was determined as the average of the oxygen uptake during the last 30 s of the test.

### Constant-speed tests

The constant-speed tests were performed using the same motor-driven treadmill and V·O_2_ procedures adopted during the maximal incremental test. The subjects performed a standardized warm-up, consisting of a 5-min run at 8 km/h followed by a 5-min light stretch. The treadmill speeds were adjusted after warm-up and the subjects ran for 6 min wearing either the TPU or minimalist shoes. Given that previous findings have shown that an athlete who is energetically economical at a given speed will not necessarily be economical at other speeds (22), the participants performed constant-speed running tests at two distinct speeds wearing either the TPE or minimalist shoes. RE was determined at 12 km/h similar to a previous study (15) and the CTO_2_ was determined at individual average speed sustained during the 3-km TT (TPU=15.6±0.8 km/h, minimalist=15.4±.6 km/h). The oxygen uptake associated with the RE and CTO_2_ was measured by averaging the last 30 s from each running constant-speed bout. Recovery time between these constant-speed running tests was 10 min.

### Running performance

The 3-km race is the official track running event that has been used by previous studies that analyzed the determinants of running performance (23,24). The participants used either the TPU or minimalist shoes during the 3-km TT, in separate sessions (i.e., second and third visits). Running performance was measured as the total time elapsed during the 3-km TT. The time to cover the 3-km TT was registered at each lap (i.e., 400 m) on an outdoor 400-m track with a manual stopwatch (model HS-1000, Casio, Japan), while the rating of perceived exertion (RPE) was reported by participants at each lap using the Borg 15-point scale (25). Copies of this scale were laminated, reduced to 10 by 5 cm and fixed to the participant's wrist on the dominant arm. Subjects were instructed to finish the race as quickly as possible, as in a competitive event. Before the test, the participants warmed up for 10 min at 8 km/h. They were instructed to maintain regular water consumption 24 h before the test and water was provided *ad libitum* during the entire event. Verbal encouragement was provided during the entire event, but runners were not advised of their lap splits. Ambient temperature and humidity were provided by the Institute of Astronomy, Geophysics and Atmospheric Sciences of the University of São Paulo, Brazil. The mean±SD values for temperature and humidity were 24±2°C and 60±8%, respectively.

### Experimental footwear

The experimental footwear used in the current study consisted of minimalist (Nike Free Run 2, average shoe mass: 275 g, heel drop: 4 mm) and TPU (Adidas Energy Boost^TM^, average shoe mass: 320 g, heel drop: 10 mm) running shoes. The minimalist model was characterized by a ultraflexible sole, lightweight, and no motion control or stability features, as previously described (26). The midsoles of the TPU model were composed of 80 and 20% of TPU and ethylene vinyl acetate, respectively. Shoe sizes ranged from 8-10 (UK system). Both shoes were wrapped with black tape to blind the participants regarding the footwear used in each experimental session. Participants had not used either of these shoes and, therefore, TPU and minimalist shoes were novel to all participants.

### Statistical analysis

A normal data distribution was confirmed by the Shapiro-Wilk test. Data are reported as means±SD. RE, CTO_2_, and running performance were compared between shoes using paired *t*-tests. Two-way ANOVA (shoes *vs* distance) was used to compare running speed distribution and RPE responses throughout the 3-km TT. Effect size (ES) was quantified using standardized mean differences and defined as trivial (<0.20), small (0.20-0.49), moderate (0.50-0.79), and large (≥0.80). A significance criterion of P<0.05 was adopted for all analyses. All statistical analyses were performed using the Statistica 8 software package (StataSoft Inc., USA).

## Results

**Figure 1:**
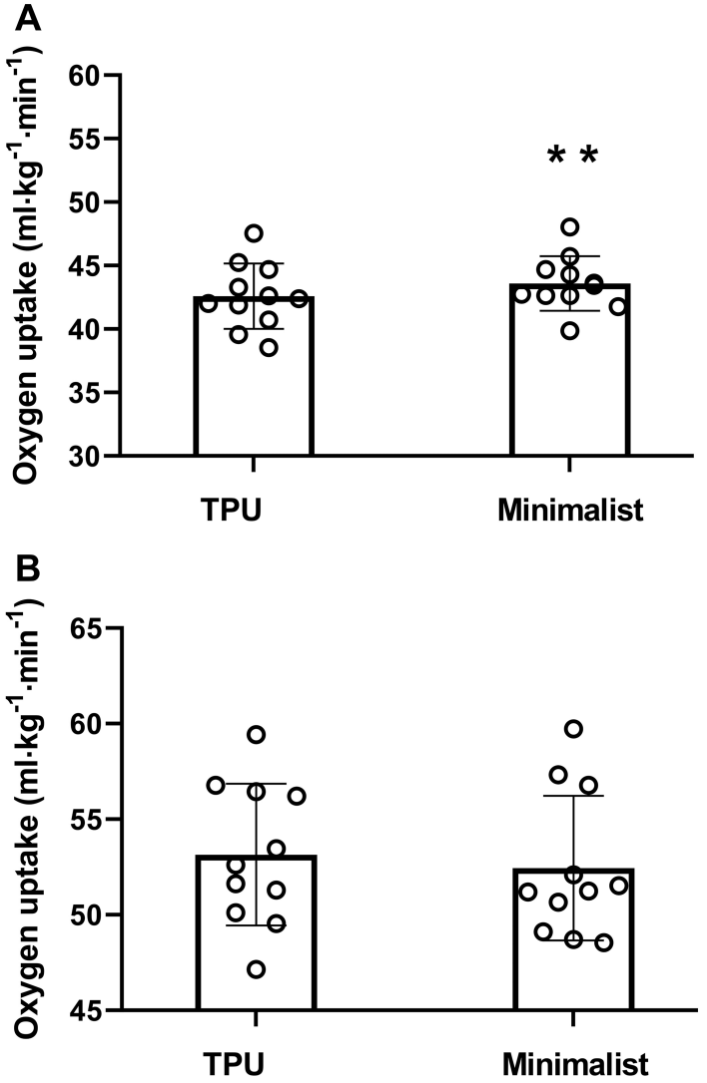
Oxygen uptake during constant-speed tests. **A**, Running economy at 12 km/h. **B**, Oxygen cost of running at individual average speed sustained during the 3-km running time-trial. TPU: midsole material composed by thermoplastic polyurethane. Data are reported as means±SD. **P<0.05 (*t*-test).

**Figure 2:**
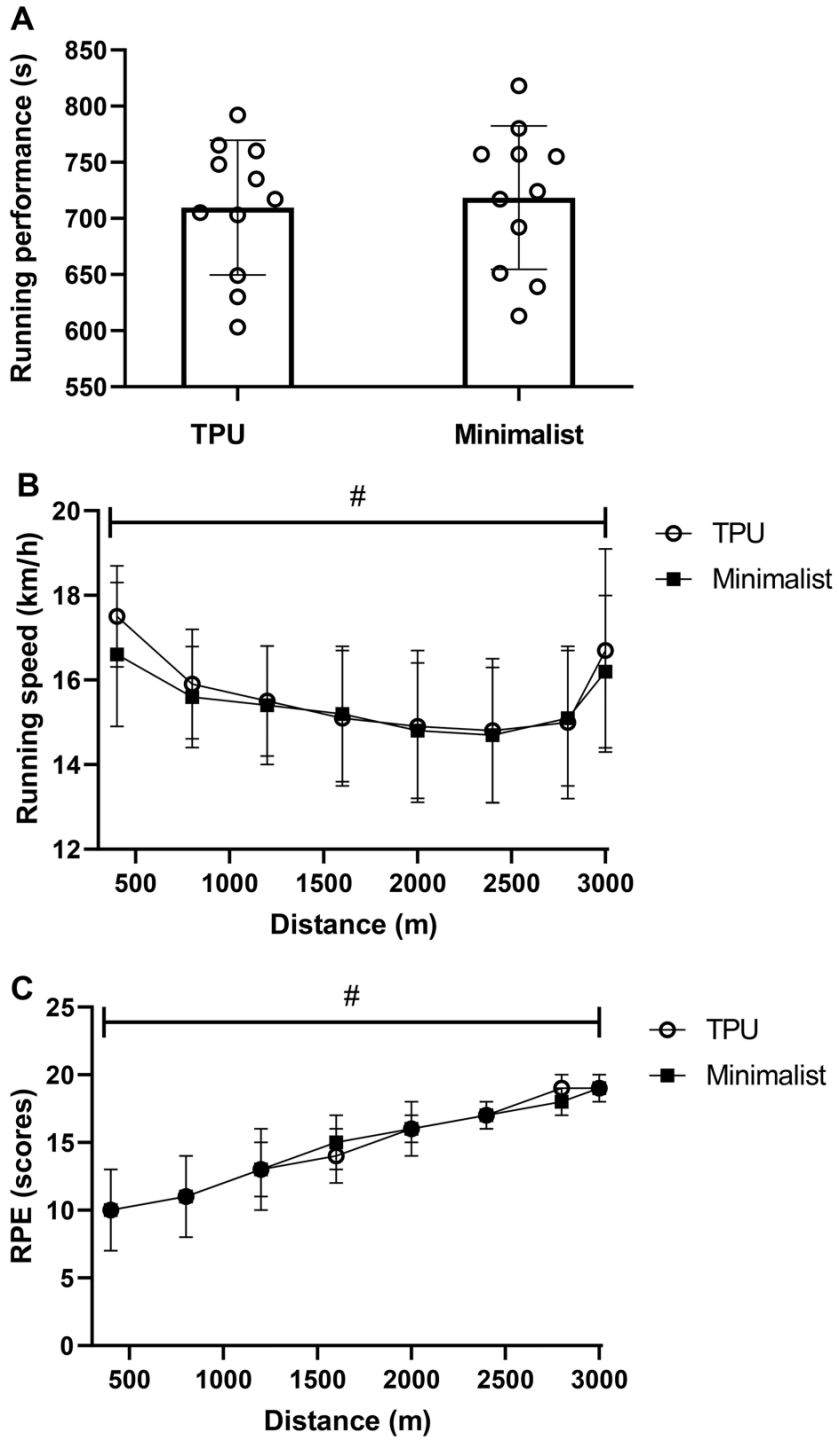
**A**, Overall performance; **B**, running speed distribution; **C**, rating of perceived exertion (RPE). TPU: midsole material composed by thermoplastic polyurethane. Data are reported as means±SD. ^#^P<0.05 for main effects for distance (*t*-test and ANOVA).

Table 1 presents the main characteristics of the participants. The RE and CTO_2_ are shown in Figure 1, while running overall performance, running speed distribution, and RPE changes throughout the 3-km TT for each shoe condition are shown in Figure 2. TPU footwear (V·O_2_=42.5±2.6 mL·kg^-1^·min^-1^) resulted in better RE (∼2.4%) compared to minimalist footwear (V·O_2_=43.6±2.1 mL·kg^-1^·min^-1^) (P=0.01, ES=0.42) (Figure 1A). In contrast, the CTO_2_ was not significantly different (P=0.61, ES=0.18) between TPU (V·O_2_=53.1±3.7 mL·kg^-1^·min^-1^) and minimalist (V·O_2_=52.4±3.8 mL·kg^-1^·min^-1^) shoes (Figure 1B). For both experimental conditions, running speed distribution showed a classical U-shaped pacing profile (Figure 2B) with the first and last laps faster than other laps, while RPE showed a linear profile (Figure 2C). However, there was a main effect only for distance (P<0.01), without main effects for shoes (P=0.67) and interaction (P=0.75) for running speed distribution. Also, there was a main effect for distance (P<0.01), but not for shoes (P=0.62) and interaction (P=0.38) for RPE. In addition, there was no statistical difference for overall running performance between footwear models (TPU shoe=701±62 s, minimalist shoe=709±61 s, P=0.52, ES=0.18) (Figure 2A).

**Table 1:** Characteristics of the runners that participated in the study.

Age (years)	33.1±7.2
Running experience (years)	4.1±2.5
Training volume (km/week)	44.7±14.3
Height (m)	1.74±0.05
Body mass (kg)	70.1±9.9
Maximal oxygen uptake (mL·kg^-1^·min^-1^)	52.1±4.9

Data are reported as means±SD.

## Discussion

Based on the assumption that oxygen cost of running is one of the best predictors of endurance performance (27,28) and that RE is acutely improved wearing both minimalist and TPU shoes (29,30), the present study aimed to compare the effects of these distinct models on RE and running performance. The main results of the current study revealed that: i) TPU shoes resulted in better RE (∼2.4%), but similar CTO_2_ compared to the minimalist shoes, and ii) there was no significant difference for running parameters (i.e., overall performance, running speed distribution, and RPE responses) between the TPU and minimalist shoes. These findings suggested that TPU shoes reduced the oxygen cost of running at low (i.e., 12 km/h) but not at high (i.e., individual average speed sustained during the 3-km TT) running speeds, resulting in a similar endurance performance compared with minimalist shoes.

Endurance running has become a very popular physical activity with millions of recreational runners starting the activity in the past few years (5). This increased popularity brought attention to the development of different training methods and technologies focused on acute improvement of endurance performance, such as a wide range of running shoe models commercially available (16,31). Considering previous studies suggesting that the low mass of minimalist models is the main characteristic that affects the energetic cost of running (14,15), in the present study the footwear mass was normalized by adding lead tape to the minimalist shoes in order to reduce the possible influence on RE, CTO_2_, and running performance. Our results showed that the footwear with TPU midsole material resulted in better RE (2.4%) compared to minimalist shoes (Figure 1). In comparison with previous findings, the changes in RE with TPU shoes was slightly below those reported by Sinclair et al. (14) who compared the TPU and minimalist shoes (∼5%), but similar to previous results by Worobets et al. (32) and Sinclair et al. (15), wherein the TPU shoes were able to improve approximately 1-4% of RE compared with conventional shoes. These findings are in accordance with the energy return advantage attributed to the TPU material, which suggested that RE could be significantly improved (15). The mechanisms by which the TPU material could improve RE are not fully understood, but it has been suggested that the TPU material within the midsole would reduce the force needed to push the ground during the propulsion phase, resulting in lower metabolic stress in active skeletal muscles of lower limbs (33). Together, these findings expand the notion that TPU could improve RE by enhanced mechanical energy return (14–16,32), even when compared with minimalist footwear.

Even though a growing amount of evidence has shown substantial gains in RE with different footwear models (7–10), there is a lack of information in the literature concerning their acute effects on endurance performance. In the present study, we analyzed the effects of TPU and minimalist shoes on a 3-km TT performance. Our results revealed that running parameters (i.e., overall performance, running speed distribution, and RPE responses) were not different between TPU and minimalist conditions (Figure 2), despite better RE shown for TPU shoes (Figure 1). The reasons for the similar running parameters despite a better RE with the TPU are not clear, but it could be related to the oxygen cost of running at the average speed at which the 3-km TT was performed. The CTO_2_ was not significantly different between the models, indicating that the TPU was not able to maintain the reduced oxygen cost during high running intensity compared to the minimalist model. This finding is novel and relevant because previous findings have demonstrated that improvements in energetic cost of running are more effective to endurance performance if observed at intensities similar to the speed performed in the actual race rather than fixed submaximal constant-speed tests (34), such as the speed chosen for the RE test (i.e., 12 km/h). Therefore, the similar running performance observed between running shoes could suggest an inability of the TPU material to maintain reduced energetic cost at running speeds similar to those adopted by athletes during 3-km TT.

In order to address a final conclusion, some limitations of the present study must be highlighted. First, the running performance was determined as the total time to cover a 3-km course, which is relatively short compared to other running events (e.g., 5-, 10-, and 21-km). Thus, given that longer running events are performed at lower relative intensities (closer to 12 km/h), it would be important to compare the effects of the footwear in longer distance running events. Second, we tested only one model of shoes designed to enhance energy return, which was composed by ∼80% of TPU in its midsole. Perhaps shoes with different percentages of TPU in the midsole (50-100% of TPU) could exacerbate the responses in oxygen cost found in the current study.

In conclusion, the findings of the current study revealed that footwear with TPU midsole material increases RE at low running speed (12 km/h) compared with minimalist shoes. However, the better RE was not evident at the average speed sustained during 3-km TT (∼15 km/h), ultimately resulting in a similar running performance compared to minimalist shoes. Therefore, it could be suggested that improved RE observed with the shoe material designed to enhance energy return could be more relevant than the minimalist nature of models for longer distance running events (≥5 km).
